# Supplementary material for: Oligosaccharyltransferase Is Involved in Targeting to ER-Associated Degradation
Source: Cells. 2025 Oct 14;14(20):1593. doi: 10.3390/cells14201593 (PMC12564220; doi:10.3390/cells14201593)
Supplement: Supplementary file 1 [file cells-14-01593-s001.zip › Shenkman Suppl Info 13-10-25.pdf]

Oligosaccharyltransferase is involved in targeting to ER-associated degradation

Marina Shenkman, Navit Ogen-Shtern, Chaitanya Patel, Haddas Saad, Bella Groisman, Metsada Pasmanik-Chor, Sonya M. Schermann, Roman Körner and Gerardo Z. Lederkremer

Supplementary Information

Supplementary figures

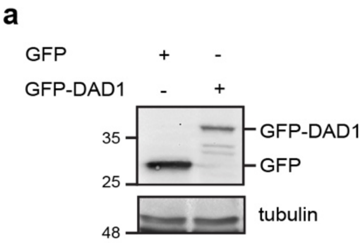

Suppl. Fig S1. Overexpression and knockdown of OST subunits. Lysates of HEK293 cells overexpressing GFP-DAD1 or GFP or after knockdown of DAD1 or Tusc3 for 48h, were immunoblotted with anti-GFP (a), DAD1 (b) and Tusc3 (c), with tubulin or actin as loading controls.

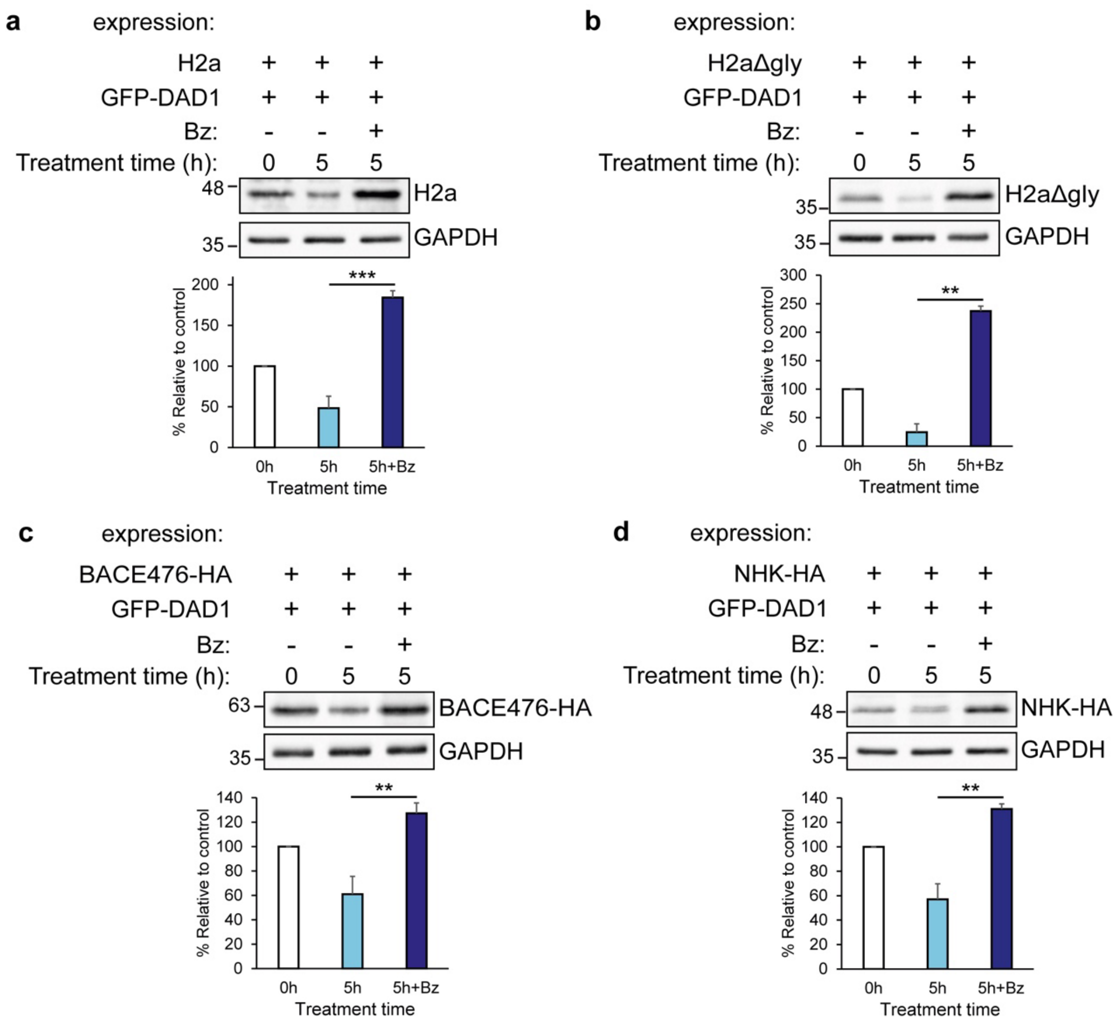

Suppl. Fig S2. Degradation caused by GFP-DAD1 is proteasome-dependent. Lysates of HEK293 cells transiently expressing H2a (H2aG78R-Myc) (a), H2aΔgly (b), BACE476-HA (c) or NHK-HA (d) and overexpressing GFP-DAD1 for 19h after transfection were treated with or without Bz (5h, 5μM). Treatment time refers to time from start of treatment. Cell lysates were immunoblotted with anti-H2a, anti-Myc and anti-HA, with GAPDH as loading control. The graphs show averages of 3 independent experiments ±SD. *P* value for: H2a ± Bz = 0.00089, *P* value for: H2aΔgly ± Bz = 0.0011, *P* value for: BACE476-HA ± Bz = 0.0053, *P* value for: NHK-HA ± Bz = 0.0015. Note the expected increase with time of expression in the presence of Bz and the decrease caused by GFP-DAD1-induced degradation in the absence of the Bz.

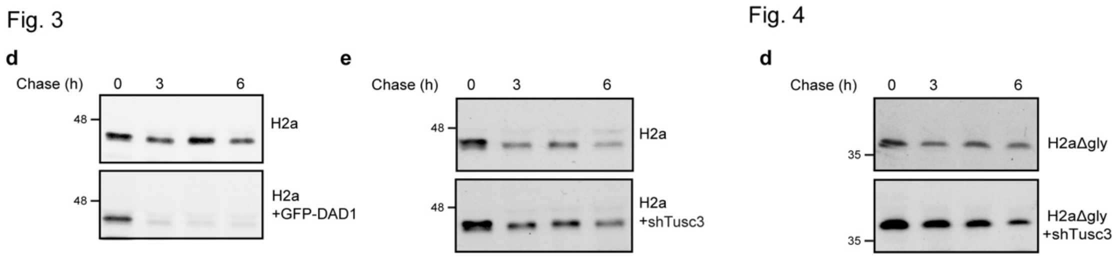

Suppl. Fig S3. Gels in Fig. 3 and 4 including uncropped lanes.

Supplementary Tables

Suppl. Table S1. OST subunits detected by mass spectrometry. LC-MS/MS analysis following the experimental scheme of Fig. 1a. Results of >2 fold labeled/unlabeled from cells treated with MG-132 and ALLN for 4h and puromycin for 15 min before lysis were compared to those from cells treated with only puromycin for 15 min, after normalization to the total amount of H2a-SBP (ASGPR2, bait) obtained. Hits are ranked by enrichment in cells treated with vs. without proteasome inhibitors. Only OST subunits, with enrichment values of 2-fold or higher in cells treated with vs. without proteasome inhibitors or found only in cells treated with proteasome inhibitors are shown. See Suppl. Tables 3 and 4 for complete lists of hits.

Suppl. Table S2. DAVID function enrichment analysis.

Suppl. Table S3. The table shows values for the 86 differentially expressed proteins resulting from the proteomics analysis.

Suppl. Table S4. Statistics analysis of the proteomics values. One list shows all proteins detected at least 3 times in 4 independent experiments. The second list shows all proteins detected at least 3 times in 4 experiments with median ratio > 1.5. The third list contains proteins detected at least 3 times in 4 experiments with median ratio > 1.5 and a student t-test p-value below 0.05.
